# Impact of interstitial impurities on the trapping of dislocation loops in tungsten

**DOI:** 10.1038/s41598-021-91390-1

**Published:** 2021-06-10

**Authors:** Alexander Bakaev, Giovanni Bonny, Nicolas Castin, Dmitry Terentyev, Viktor A. Bakaev

**Affiliations:** 1 SCK CEN, Nuclear Materials Science Institute, Boeretang 200, 2400 Mol, Belgium; 2 Institute of Physics, Nanotechnology and Telecommunications, Peter the Great St. Petersburg Polytechnic University, 29 Polytekhnicheskaya str, St. Petersburg, Russia 195251

**Keywords:** Electronic properties and materials, Structure of solids and liquids, Electronic structure of atoms and molecules, Thermodynamics

## Abstract

Ab initio simulations are employed to assess the interaction of typical interstitial impurities with self-interstitial atoms, dislocation loops and edge dislocation lines in tungsten. These impurities are present in commercial tungsten grades and are also created as a result of neutron transmutation or the plasma in-take process. The relevance of the study is determined by the application of tungsten as first wall material in fusion reactors. For the defects with dislocation character, the following ordering of the interaction strength was established: H < N < C < O < He. The magnitude of the interaction energy was rationalized by decomposing it into elastic (related to the lattice strain) and chemical (related to local electron density) contributions. To account for the combined effect of impurity concentration and pinning strength, the impact of the presence of these impurities on the mobility of isolated dislocation loops was studied for DEMO relevant conditions in the non-elastic and dilute limit.

## Introduction

Tungsten (W) is an important material for thermonuclear fusion reactor applications. It has been selected as diverter armor material in ITER and is a candidate material for plasma-facing components in future projects, such as DEMO^1,2^. Under operation, these components will degrade under the influence of harsh neutron irradiation, which induces microstructural damage in the material.

Dislocation loops are typical ion or neutron irradiation-induced defects in W, which are the prevailing defects at irradiation temperatures below 800 °C and doses below 1 dpa^3^. The evolution of the irradiation-induced microstructure, including the nucleation and growth of dislocation loops, is a very complex phenomenon. In pure (impurity-free) W, the vast majority of the formed dislocation loops are of ½ < 111 > type, which are able to migrate very fast even at low temperatures (room temperature and above), due to a very low migration activation barrier (~ 0.01 eV^4^), if no other pinning defects are present or their density is low. The experimental results of ion^5,6^ and neutron^7^ irradiations up to 4 dpa of various tungsten grades at temperatures from RT (room temperature) to 1400 °C have pointed to a high complexity of the developed dense microstructure, which included both transmission electron microscopy (TEM) visible and invisible damage. Such a microstructure gradually emerging with increasing dose will inevitably contribute to obstruction of the mobility of dislocation loops.

A typical industrial W grade^8^, although being very pure (of 99.97 wt% purity as in the case of the Plansee W grade), contains a certain amount of impurity atoms, including interstitial ones, such as C, H, He, N and O, which are in focus of this work. To illustrate this, the impurities specification sheet of a Plansee W grade^8^ is summarized in Table 1. Under the influence of neutron irradiation, non-negligible amounts of H and He isotopes are generated in bulk W via nuclear reactions. In the work by Gilbert et al.^9^, concentrations of interstitial impurities equal to 76 appm H and 34 appm He were estimated in bulk W after 5 years of full power operation in DEMO due to transmutation processes. In addition to these two interstitial impurities a much larger concentration^9^ of substitutional impurities will be created already after 1 full power year including 9060 appm of Re and 558 appm of Os.

**Table 1:** The list of interstitial impurities contained in a W grade manufactured by Plansee^8^.

Impurity	Concentration (in appm) according to the manufacturer		Comment
	Typical	Guaranteed maximum	
C	92	458	The most abundant interstitial impurity in W. It is often injected during ion irradiation^10,11^
H	0	911	They are accumulated as a result of plasma-related high-flux low-energy hydrogen and helium ion implantation as well as via nuclear transmutation reactions
He	0	183 (if all ‘other’ impurities from the specification table are He atoms)	
N	13	66	It is often injected during ion irradiation^11^
O	23	229	

The importance of accounting for the presence of these impurities, which serve as traps for mobile irradiation defects, has been illustrated in several kinetic Monte Carlo studies, where the strength and concentration of traps were shown to have a significant impact on the evolution of the irradiation-induced microstructure^12–16^. Specifically, it was shown that increasing the C concentration impedes the elimination of loops at sinks (dislocations and grain boundary), and reduces the rate of coarsening of loops in the bulk. The direct consequences of these effects are that, at a given dose, the number density of loops observed in the bulk increases, and their average size decreases. Furthermore, since the rate of recombination with vacancy defects is affected, the number density of voids formed in the bulk decreases.

Another important drive for the knowledge of impurity-defect interaction is the usage of ion beam accelerators to perform high damage level/rate studies surrogating neutron irradiation^10,11^. It is relatively well established by now that ion/electron irradiation may lead to the surface contamination by oxygen^11^, carbon^10,11^ and nitrogen^11^, which in turn will affect the evolution of the microstructure under irradiation.

So far, only limited data has been published on the interaction of typical interstitial impurities such as C, H, He, N and O with self-interstitial atom (SIA) clusters and dislocation loops. The interaction of C with single SIAs and edge dislocations (the limiting case for a loop of infinite size) has been addressed in our recent work^17^, where a strong interaction energy of − 0.7 and − 2.1 eV has been reported, respectively. The interaction energy of H and He atoms with SIAs was computed in the study by Becquart et al.^4^, where an attraction of − 0.3 and − 0.9 eV was found, respectively. Another study^18^ has shown that H and He atoms are strongly bound to an edge dislocation with the interaction energy of − 0.9 and − 3.0 eV, respectively. In the work by De Backer et al.^19^, the interaction of H isotopes with SIA clusters and dislocation loops up to 37 SIAs was addressed, where an interaction energy in the range of − 0.3 to − 0.7 eV was derived. Thus, no information on the interaction of C, O, N and He with SIA clusters (dislocation loops) and of O and N to edge dislocations has been published so far.

Therefore, in the present work, we perform a systematic study to derive the interaction strength of the typical interstitial impurities C, H, He, N and O, contained in commercial tungsten grades and irradiation-induced defects such as SIA clusters and dislocation loops. Density functional theory (DFT) computations are employed to characterize the interaction energy between each impurity and an SIA cluster (dislocation loop) of sizes 7, 19 and 37. As limiting case for a loop of infinite size, a ½ < 111 > edge dislocation was used. The observed interaction energy for the different impurities is rationalized based on elastic interactions with the loop’s strain field and chemical bonding contribution via charge density maps and bond strength analyses.

To understand the effect of each impurity on the loop mobility and hence irradiation microstructure, the combined effect of both trapping strength and impurity concentration must be addressed. To do this, the effective diffusion coefficient of a single 1-D migrating loop in a dilute field of impurities is computed, based on the DFT data. As such, depending on their concentration and interaction strength, the most impacting interstitial impurities on the irradiation-induced microstructure are identified at the beginning and after 5 years of DEMO operation.

## Methods

The applied methodology can be described as follows. The computational analysis was carried out performing density functional theory (DFT) calculations with the Vienna Ab initio Simulation package (VASP)^20,21^ utilizing the projector-augmented wave (PAW) pseudopotentials^22,23^. The generalized gradient approximation with the parameter set by Perdew, Burke and Ernzerhof (PBE)^24^ was applied for the treatment of exchange–correlation effects. The atomic configuration was non spin-polarized (non-magnetic). The pseudopotentials with six, four, five, one, two and six valence electrons were employed to describe W, C, N, H, He and O, respectively. Ionic relaxation was realized using the conjugate gradient algorithm with a force convergence criterion equal to 0.03 eV/Å. The relaxation of the electronic subsystem was performed with a global break condition of 10^−4^ eV. A Methfessel-Paxton smearing of 0.3 eV has been employed.

The atomic configurations containing an impurity in the bulk W, an SIA and impurity with SIA were assessed using the simulation box of the size 4 × 4 × 4 a_0_^3^ with crystallographic orientations [100] × [010] × [001], where the lattice parameter *a*_*0*_ is equal to 3.172 Å. The k-point mesh of 3 × 3 × 3 was employed for the system that consisted of 128 atoms prior to insertion of an SIA or impurity. For the impurity-SIA interaction analysis, firstly all non-equivalent tetrahedral and octahedral positions for impurities around the SIA defect, inserted in the box and oriented along the direction < 11> (the lowest energy SIA configuration in W^25^) were scanned to identify the lowest energy SIA-impurity configuration. Later on, these selected lowest-energy atomic structures were relaxed in a larger box of size 5 × 5 × 5 a_0_^3^ (with the k-point mesh equal to 5 × 5 × 5) to obtain the converged values for the interaction energy following our previous study^25^.

The dislocation loops were studied using two simulation boxes, namely 8 × 8 × 8 (1024 atoms) and 9 × 9 × 9 *a*_*0*_^3^ (1458 atoms) with crystallographic orientations [100] × [010] × [001]. Three different dislocation loops with a Burgers vector (BV) ½ < 111 > were introduced in the bulk W crystal. Perfect hexagonal loops were created by introducing <111> self-interstitial atoms (SIA) such that clusters containing 7, 19 and 37 SIAs were created, corresponding to loops with 0.7, 1.2 and 1.7 nm in diameter, respectively. The loop diameter *d* was calculated using the formula $d = 2a_{0} \sqrt {\frac{m}{\sqrt 3 \pi }}$, where *m* – is the number of SIAs forming the dislocation loop as proposed by De Backer et al^26^. The crystals with the dislocation loops were relaxed prior to insertion of the impurities.

A dedicated convergence test with two different simulation box sizes was performed. The following k-point meshes were tested to identify the dislocation loop-impurity interaction strength: 1 × 1 × 1 Gamma point, Monkhorst–Pack: 1 × 1 × 1, 2 × 2 × 2 and 3 × 3 × 3 (the largest possible given the available supercomputer memory limitations). Based on the outcome of this test, the converged results reported in the paper were obtained using the Monkhorst–pack k-point meshing of 2 × 2 × 2 in a 9 × 9 × 9 simulation box. The details of this convergence study are reported in the Supplementary Material: Appendix A.

**Figure 1:**
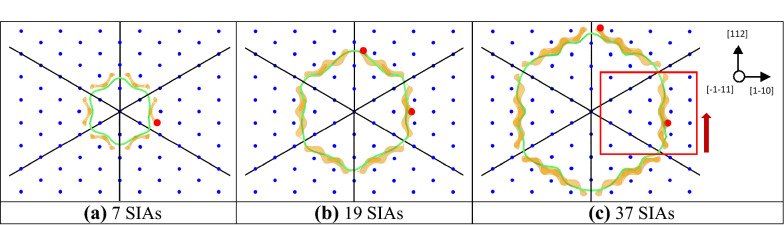
Charge density maps for the three dislocation loops considered in this study. The green line shows the position of dislocation line of the dislocation loop identified using OVITO with DXA method. The orange isosurfaces correspond to a charge density equal to 9.03 × 10^–6^ e/Å^3^. The red points reveal the different initial positions for the impurity. The red square highlights the zone (perpendicular to the graph’s plane) used for an in-depth analysis in the results section, while the red arrow shows the corresponding viewing direction. The images are created with OVITO [‘https://www.ovito.org/’ version 3.0.0-dev]^27^*.*

In order to assess the dislocation loop-defect interaction energy, a single interstitial atom (C, N, H, He or O) was inserted into one of two positions for each of the loop sizes (except for the smallest loop, where a single position was considered): in the center of an edge and at the corner. The exact positioning was identified on the basis of the lowest charge density zones of the relaxed atomic configuration with the relevant dislocation loop (see Fig. 1). The visualizations of atomic structures and charge density isosurfaces shown in this figure and in the following were created using the OVITO visualization software^27^. The dislocation lines positions and their length $L$ were obtained using the Dislocation Extraction Algorithm (DXA) tool^28^ embedded in OVITO.

The interaction of impurities, such as N and O with edge dislocations was assessed in a similar way as in our previous papers, where the interaction of H, He and C with an edge dislocation was studied^25,29^. For detailed information and convergence studies we refer the reader to these papers, while here we provide a brief outline. The simulation box with 1-D-periodicity and with fixed boundary conditions in the *xy* plane was created contained a single edge dislocation with BV = ½ < 111 > . Its dimensions were 24.82 × 7.78 × 22.51 Å^3^ in the [− 1–11] (*x*), [1–10] (*y*), and [112] (*z*) orientations, respectively, containing a total of 246 W atoms.

The interaction energy between the defect (SIA, SIA cluster or edge dislocation) and the impurity was calculated following,

1 $$E_{i} = \left( {E_{defect \& impurity} + E_{pure} } \right) - \left( {E_{defect} + E_{impurity} } \right),$$

where $E_{defect \& impurity}$ is the total energy of the simulation box with the defect-impurity cluster, while $E_{defect}$, $E_{impurity}$ and $E_{pure}$ are the total energy of the crystals with the defect, impurity only or defect-free, respectively. Following this notation, a negative interaction energy implies an attractive interaction and vice versa.

For the interaction of impurities with single SIAs and SIA clusters, the elastic correction by Varvenne et al.^30^ was applied to the total energy of the crystals. In this approach ab initio calculations and linear elasticity theory are coupled to obtain the correct energetics of an isolated defect cluster by compensating its large strain fields interacting through the periodic boundaries. It required as input data the stress tensor of the resulting configurations and the elastic constants of the material. The latter were calculated in a bcc box containing two atoms with k-point mesh 21 × 21 × 21 and are presented in Supplementary Material: Appendix B.

In order to rationalize the interaction energy between the considered interstitial impurities and dislocation loops we have decomposed the value of this energy into the elastic and electronic (or chemical) contributions following the methodology proposed by De Backer et al.^26^. The elastic contribution was assessed,

2 $$E_{i}^{el} = \left( {E_{loop + impurity} - E_{loop + impurity}^{no\,impurity} } \right) - \left( {E_{impurity\,in\,bulk} - E_{impurity\,in\,bulk}^{no\,impurity} } \right),$$

where $$E_{loop + impurity}^{no impurity}$$ is the total energy of the relaxed atomic configuration of the impurity next to the dislocation loop, but without the impurity present, calculated after electronic relaxation only (i.e. no ionic relaxation was allowed). In a similar way, $$E_{impurity in bulk}^{no impurity}$$ is the total energy of the distorted W lattice after removal of the impurity from its lowest energy tetrahedral or octahedral position (whichever is lower) after electronic relaxation only. The chemical contribution to the impurity-loop interaction energy was finally calculated as the difference of the total interaction energy and the corresponding elastic contribution.

The elastic contribution to the total energy was further explored by correlating it with the magnitude of minimization of relaxation volume of the impurities upon allocation at the dislocation loop as compared to the bulk material. The relaxation volume provides an information on the extra volume introduced by the defect after its insertion in the W lattice. The relaxation volume $\Omega$ was calculated using the algorithm and script provided in the work by Varvenne et al.^30^.

The chemical contribution to the total energy was assessed on the basis of charge density difference maps and bond strength analysis. The charge density difference maps for the chemical environment of impurities in bulk W and in the vicinity of dislocation loops were created following,

3 $$\Delta \rho = \rho_{W environment \& impurity} - \rho_{W environment} - \rho_{impurity} ,$$

where $\rho_{W environment \& impurity}$ is the charge density of the relaxed crystal with the impurity at the dislocation loop or in bulk W, while the charge densities $\rho_{impurity}$ and $\rho_{W environment}$ were obtained by performing the electronic relaxation of the same crystal with the impurity only or W environment, such as dislocation loop or bulk W (distorted due to presence of the impurity), respectively.

The chemical bond analysis for the various impurities in bulk W and in the vicinity of dislocation loops was realized using the density derived electrostatic and chemical (DDEC) method with the charge partitioning version 6^31–33^. This method implemented into the Chargemol program^34^ provides the information on the net atomic charges as well as bond orders and the sums of the latter for every atom (referred below as SBO). This allows one to extract the data on the bond character (covalent, polar-covalent or ionic) and quantify the change of the bond strength^33^ for the impurities undergoing the migration from the bulk to the dislocation loop.

## Results

**Figure 2:**
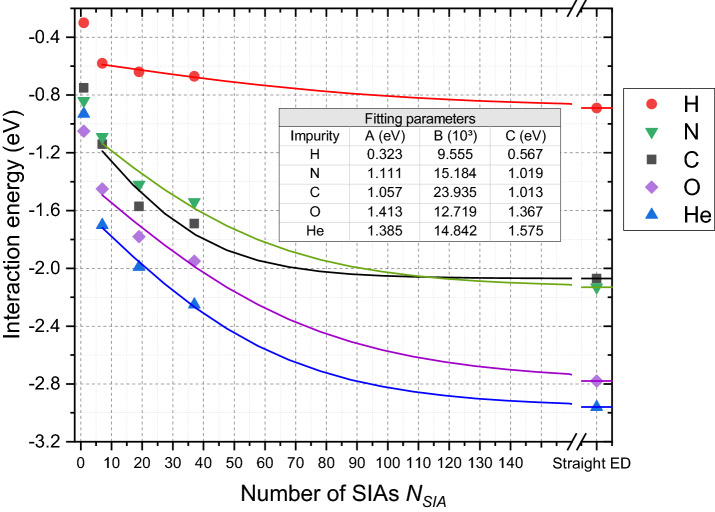
Interaction energy of interstitial impurities (H, N, C, O and He) with interstitial defects (single SIA, dislocation loops of various sizes (with 7, 19, 37 SIAs) and edge dislocation) in bcc W. The lines reproduce the resulting fitted curves for the interaction energy for each of the impurities. The inline table reports the fitting parameters for the fitted curves.

The results of our ab initio simulations, namely the interaction energy between the interstitial impurities H, N, C, O and He, and dislocation loops of three sizes studied in this work is summarized in Table 2. The interaction energy for the impurities in the lowest-energy position next to the dislocation loops along with the SIAs and edge dislocations is visualized in Fig. 2. It is seen clearly that the increase of the size of the loop or, in other words, number of SIAs leads to the increase of the interaction strength for any of the impurities. Table 2 also clarifies that all the impurities tend to prefer the lowest-charge density positions at the middle of the edge of the loop, rather than at the corner of it, except for the smaller loop with 17 SIAs. For the latter case, there is no difference (< 0.01 eV) for the He atom to reside at the corner or at the edge of the loop. The elastic correction was found to be important for obtaining the correct converged values of the interaction energy. While the small dislocation loop of 7 SIAs introduces a moderate strain field in the relatively large crystal (9 × 9 × 9) considered in this work, the two larger loops of 17 and 37 SIAs have a large strained zone, thus the interaction energy was corrected by up to 0.15 and 0.31 eV, respectively.

**Table 2:** Interaction energy between dislocation loops and impurities in different positions with (EL) and without (NEL) elastic correction. The difference of these values (${\Delta }$) is also presented.

Impurity	Loop-impurity interaction energy (eV)															
	7 SIA			17 SIA							37 SIA					
	Edge			Edge			Corner				Edge			Corner		
	NEL	EL	${\Delta }$	NEL	EL	${\Delta }$	NEL		EL	${\Delta }$	NEL	EL	${\Delta }$	NEL	EL	${\Delta }$
H	− 0.56	− 0.58	− 0.02	− 0.60	− 0.64	− 0.04	− 0.57		− 0.61	− 0.04	− 0.58	− 0.67	− 0.09	− 0.54	− 0.64	− 0.10
N	− 1.03	− 1.09	− 0.06	− 1.29	− 1.42	− 0.13	− 1.14		− 1.28	− 0.14	− 1.29	− 1.54	− 0.25	− 1.03	− 1.32	− 0.29
C	− 1.07	− 1.14	− 0.07	− 1.45	− 1.57	− 0.13	− 1.19		− 1.33	− 0.14	− 1.43	− 1.69	− 0.25	− 1.09	− 1.39	− 0.30
O	− 1.39	− 1.45	− 0.06	− 1.64	− 1.78	− 0.14	− 1.56		− 1.71	− 0.15	− 1.66	− 1.95	− 0.29	− 1.42	− 1.73	− 0.31
He	− 1.66	− 1.70	− 0.04	− 1.89	− 1.99	− 0.10	− 1.89		− 1.99	− 0.10	− 2.02	− 2.25	− 0.23	− 1.87	− 2.11	− 0.24
				Loop-impurity interaction energy difference for side and corner positions (eV)												
				17 SIA							37 SIA					
				NEL				EL			NEL			EL		
H				− 0.03				− 0.03			− 0.05			− 0.04		
N				− 0.16				− 0.14			− 0.26			− 0.22		
C				− 0.26				− 0.24			− 0.35			− 0.30		
O				− 0.08				− 0.07			− 0.24			− 0.22		
He				0.00				0.01			− 0.16			− 0.14

It was found that the trends in the interaction energy for various impurities are similar and weakly depend on the number of SIA in the defect. All the interstitial impurities show attraction to the tensile regions formed in the neighborhood of SIAs, dislocation loops and edge dislocations. He and O atoms reveal the strongest attraction, while H shows the weakest attraction. For C and N atoms, the interaction strength is located in between H and He or O.

The results shown in Fig. 2 are enhanced with the trend curves with the best function found interpolating the results of interaction of impurities with the defects of an edge dislocation character (dislocation loops and edge dislocations), i.e. excluding the single SIA defect. The interaction energy between impurity and loop decreases to a constant value with loop size, which in the limit should correspond to the interaction between an impurity and straight edge dislocation line. Clearly, two regimes must exist: for small loops, the interaction energy must evolve with the number of SIAs the cluster, as the impurity interacts with all SIAs in the cluster. For larger clusters, with a clear dislocation character, the value must saturate fast as the interaction is restricted to a dislocation segment (here characterized by interaction energy with an edge dislocation). As a function describing this bi-linear behavior, we propose the hyperbolic tangent function with three independent variables which can adequately describe the interaction energy trends for the studied impurities,

4 $$E_{i}^{fit} = - A\tanh \left( {BN_{SIA} } \right) - C,$$

where $N_{SIA}$—is the number of SIAs constituting the dislocation loop and $A$, $B$ and $C$ are the fitting coefficients, shown in the inline table of Fig. 2. One can derive from this fitting that starting from $N_{SIA}$. equal to 100–120, which corresponds to the dislocation loops larger than 2.7–3.0 nm in diameter, the impurity-loop interaction energy reaches the converged values close to impurity-edge dislocation interaction strength.

**Figure 3:**
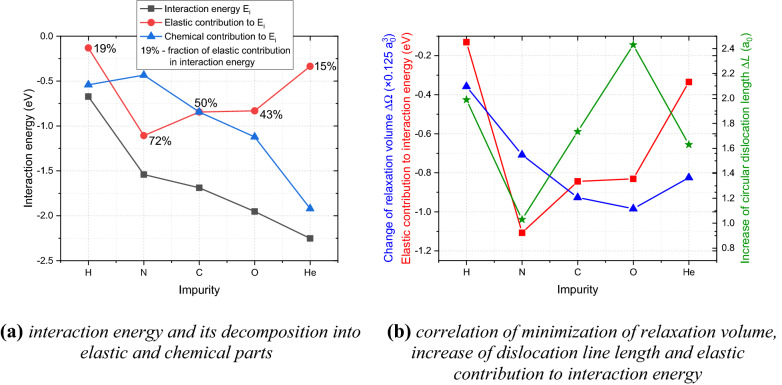
Elastic and chemical contributions to the interaction energy (**a**) and correlation of elastic contribution, minimization of relaxation volume ${{\Delta \Omega }}$ and increase of dislocation length ${\Delta L}$ (**b**) for various impurities at a dislocation loop of 37 SIAs in bcc W.

In order to rationalize the different interaction strength between the studied impurities and dislocation loops focusing on the largest dislocation loop studied here (i.e., made of 37 SIAs), we have decomposed the interaction energy into elastic (related to minimization of crystal lattice deformation (distortion) upon insertion of the impurity next to the loop as compared to the bulk crystal) and chemical (related to minimization of electronic structure effects or chemical bonding) contributions. Figure 3a illustrates that the ratio of these contributions differs significantly for these impurities. For the two lightest elements, i.e., H and He atoms, the fraction of elastic contribution barely reaches one fifth of the interaction energy value, while for the N atom almost three quarters of the interaction energy is related to a smaller lattice misfit introduced by this impurity at the dislocation loop as compared to an interstitial configuration in bulk W. In order to quantify the reduction of lattice misfit for different impurities upon reallocation from the bulk material to the tensile region of dislocation loops with a larger interatomic spacing firstly we have calculated the reduction of relaxation volume $\Delta \Omega$ for the considered interstitial impurities. As expected, it was found a good correlation between it and the elastic contribution (except for N) as shown in Fig. 3b. Then, we noted that all the studied impurities, if reallocated to the vicinity of a dislocation loop from the bulk, induce an increase of the dislocation length $\Delta L$ (i.e. slightly enlarge the dislocation loop size) as quantified in Fig. 3b. This phenomenon, unlike reduction of relaxation volume, has a negative impact on the value of the thermodynamic force (interaction energy) for an impurity to be allocated in the vicinity of dislocation line. The lowest increase of the dislocation line length and consequently the lowest energy cost related to it^35^ is observed for a N atom. This observation can eventually explain its largest elastic contribution to interaction energy as compared to other impurities.

**Figure 4:**
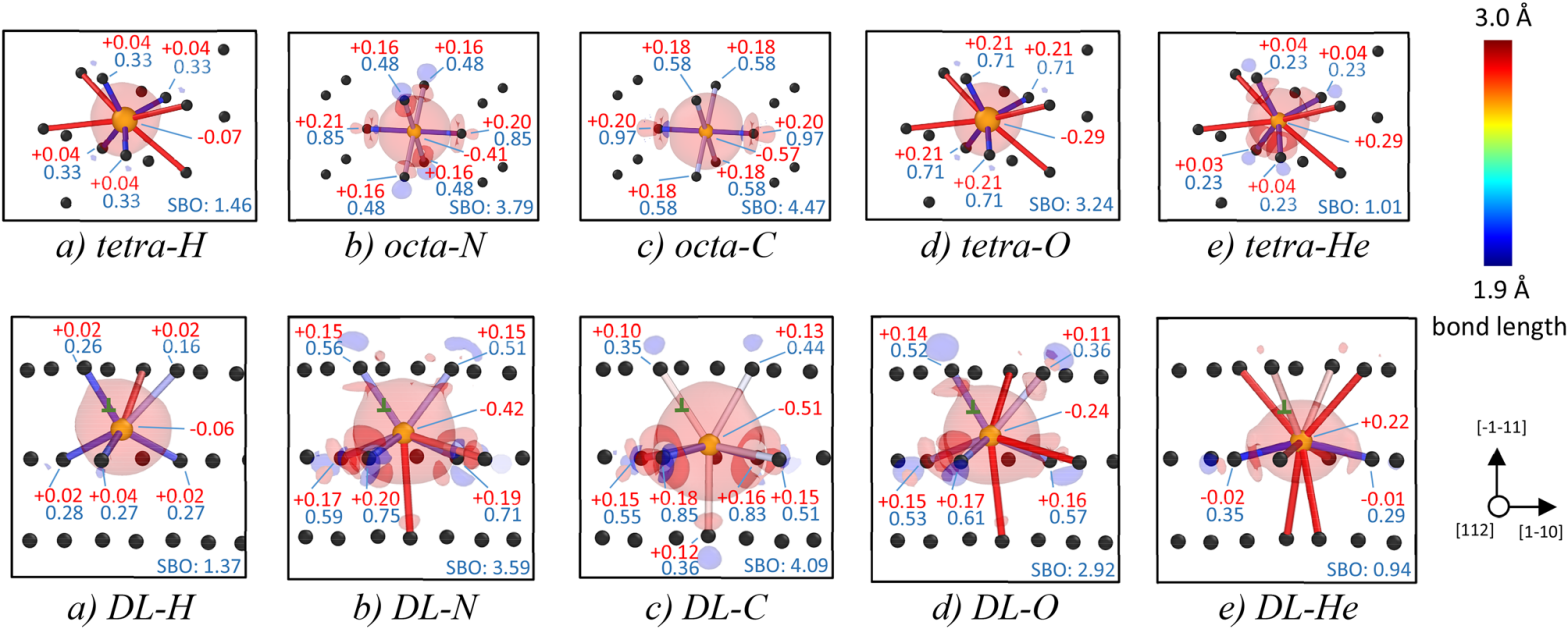
Charge density difference maps for impurities in the lowest energy position in bulk W (tetrahedral or octahedral positions) and around a dislocation loop of 37 SIAs. The view for the latter is shown in Fig. 1c. The colour of the bond corresponds to its length and is explained in the legend on the right hand side. Black circles are W atoms around the impurity, while yellow enlarged atoms are the corresponding impurities (ordered according to the their interaction energy with the loop from the lowest to the highest). The numbers in red are the corresponding net atomic charges, while the numbers in blue reveal the magnitude of bond order for the bond between the impurity and the relevant atom. The isosurfaces with the charge density difference of + 4.3 * 10^–7^ e/A^3^ (local increase of charge density) and − 4.3 * 10^–7^ e/A^3^ (local decrease of charge density) are visualized with red and blue colors, respectively. The images are created with OVITO [‘https://www.ovito.org/’ version 3.0.0-dev]^27^.

The chemical contribution to the interaction energy can be analyzed on the basis of the charge density difference maps for the interstitial impurities in the lowest energy positions (tetrahedral for H, O and He; and octahedral for N and C) as compared to the atomic configuration dislocation loop (37 SIAs)-impurity as visualized in Fig. 4. The impurity-nearest W atoms bonds are also highlighted in Fig. 4 along with the information on the net charge on these atoms and bond order values. It is clear that the absolute value of the chemical part of the interaction energy value is related to the minimization of the chemical bonding: the SBO value regularly decreases by 0.1–0.4 and the magnitude of charge induced on the impurities is reduced by up to about 0.1 *e*^*−*^ for all the considered interstitial impurities as a result of their allocation at the vicinity of the dislocation loop. In other words, the strongest bonds between impurities and nearest W atoms undergo a significant change as a result of a migration from the bulk material to the dislocation loop. No change, though, in the type of the chemical bonding is observed: all the bonds shown in Fig. 4 have a weak or strong covalent character, characterized by a very low atomic charge (below 0.3 *e*^*−*^) and significant values for the bond order (above 0.1). For the weakest chemical contribution to the interaction energy, i.e. for the case of a H atom, an increase of the strongest bonds from four to five is noted, while the number of strongest bonds is halved (from four to two) for a He atom, which is the case of the largest minimization of chemical bonding and, consequently, the largest chemical contribution to the interaction energy among the studied impurities.

## Discussion

In the previous section the results on the interaction strength between a loop and a single impurity were presented. However, as shown in Table 1, not all impurities are present in equal amounts in typical W grades. In the following, we address the combined effect of impurity concentration and trapping strength by computing their combined effect on the diffusion coefficient of the dislocation loops. By definition, the diffusion coefficient is calculated as,

5 $$\begin{array}{*{20}c} {D = \frac{{R^{2} }}{2nt},} \\ \end{array}$$

where $R^{2}$ is the mean squared displacement over the time *t* and *n* is the dimensionality of this migration equal to one in our case. In defect-free W, the diffusion coefficient of a 1-D migrating loop with Burgers vector ***b*** = ½ < 111 > is given as,

6 $$\begin{array}{*{20}c} {D_{free} = \frac{{fb^{2} }}{{2\nu_{0}^{ - 1} \exp \left( {\beta E_{m} } \right)}}, } \\ \end{array}$$

where $f$ is the correlation factor (equal to one for purely random diffusion), $\nu_{0}$ is an attempt frequency, $\beta$ = $\frac{1}{{k_{B} T}}$ (Boltzmann constant $k_{B}$ = 8.617 eV/K and T—material’s temperature) and $E_{m}$ is the loop migration energy.

In a field of static (i.e. immobile) traps, in the dilute limit (i.e. spacing between traps is much larger than the loop-trap interaction distance), such that only a single (not bound to any other defects in the crystal) impurity interacts with a loop at any given moment, the effective diffusion coefficient is given as,

7 $$\begin{array}{*{20}c} {D_{eff} = \frac{{L_{traps}^{2} }}{{2\left( {t_{free}^{*} + t_{traps} } \right)}} = \frac{{L_{traps}^{2} }}{{2\left( {\frac{{L_{traps}^{2} { }\nu_{0}^{ - 1} \exp \left( {\beta E_{m} } \right)}}{{b^{2} }} + \nu_{0}^{ - 1} \exp \left[ {\beta \left( { - E_{i} + E_{m} } \right)} \right]} \right)}},} \\ \end{array}$$

where $L_{traps}$ is the mean trap spacing, $t_{free}^{*}$ is the time for the dislocation loop to travel the me squared distance of $L_{traps}^{2}$ in the trap-free matrix given the diffusion coefficient $D_{free}$ (see Eq. 6) and $t_{traps}$ is the average time for a dislocation loop to detrap from an impurity given the dissociation energy of $( - E_{i} + E_{m} )$. The mean free path between the traps $L_{traps}$ depends on both SIA defect cluster size and trap concentration and is given^36^ as,

8 $$\begin{array}{*{20}c} {L_{traps} = \frac{1}{{\sqrt 2 \pi R^{2} N}},} \\ \end{array}$$

where $R = R_{loop} + R_{impurity}$ is the capture radius between the loop and the impurity and *N* is the number density of the latter in bulk tungsten. Here, $R_{loop}$ and $R_{impurity}$ are assumed to be equal to the half of the loop diameter and $1.5 a_{0}$, respectively.

Thus, the relative change of the diffusion coefficient for dislocation loops in the presence of the impurities in the dilute limit, $D_{eff} /D_{free}$, can be evaluated as,

9 $$\begin{array}{*{20}c} {\frac{{D_{eff} }}{{D_{free} }} = \frac{1}{{1 + \frac{{b^{2} }}{{L_{traps}^{2} }}\exp \left( { - \beta E_{i} } \right)}}.} \\ \end{array}$$

**Figure 5:**
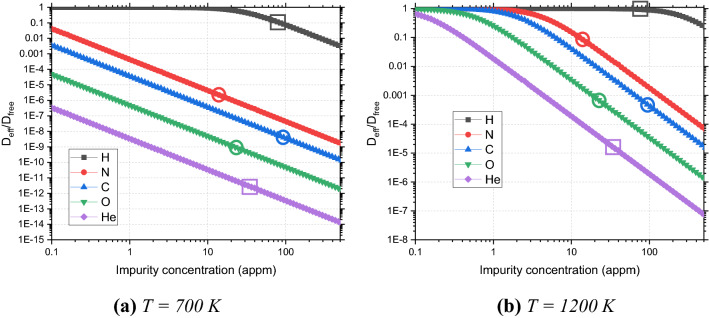
Relative change of diffusion coefficient ${\text{D}}_{{{\text{eff}}}} /{\text{D}}_{{{\text{free}}}}$ of dislocation loops with 37 SIAs in the field of different impurities depending on their concentration at the temperature equal to (**a**) 700 K and (**b**) 1200 K. The circles show the typical concentration of the impurities in the as-received state if their initial typical concentration is non-zero. The squares show the expected production of H and He due to transmutation after 5 years of operation of plasma-facing W-made components in DEMO.

In Fig. 5, the ratio $D_{eff} /D_{free}$ for the dislocation loop of 37 SIAs (i.e. of 1.7 nm in diameter) is visualized for the limiting operational temperatures of W in the range 700 K and 1200 K for different impurities, depending on their concentration. Typical concentrations of the impurities N, C and O, which are non-zero in the as-received state, are also indicated in this figure.

For both temperatures it is clear that H forms the weakest effective trap for dislocation loops, which affects the loop’s diffusion only at relatively large concentrations above 20 and 100 appm at 700 and 1200 K, respectively. For the strongest attractant to the dislocation loop, i.e. for the He impurity, even at very low concentrations 0.1–0.3 appm it has a significant impact on the loop’s diffusion. The attraction of He to the dislocation loop (and vice versa) is so strong, that if He has a concentration even 10 times lower than any of other interstitial impurities, it will still have a much stronger effect or at least comparable effect on the loop diffusion as all other interstitial impurities. Also, it can be seen that all other impurities, such as C, N and O significantly slow down the loop migration at low temperatures, such as 700 K, even at very small concentrations like 0.1 appm. For the higher temperature of 1200 K, the effect of the presence of this impurities on the loop diffusion can be noted for impurity concentrations above 0.2, 1 and 2 appm for O, C and N, respectively.

Since the evolution of impurity content in the bulk material while operation depends on many conditions, such as exposure to plasma, neutron transmutation for neutron irradiation and even injection of carbon for the special case of ion irradiation, it is difficult to predict reliably the concentration of all these five impurities at a certain time of operation. We know, however, that the typical content of all these impurities in the as received condition (see Table 1), i.e., at the beginning of the operation. Considering the limiting case such that all impurities are randomly allocated in the material (i.e. are not segregated at sinks), then the effect of their presence on the diffusion will be as shown in Fig. 5a,b by the colored circles. Clearly, the effect of N is negligible as compared to the effect of C and O on the loop diffusion. For both considered temperatures, the effect of the latter is of the same order, which implies that given their typical initial concentration, both C and O atoms are equally responsible for the slowing down of loop diffusion and can both prevent the loops from disappearance at sinks, such as the dislocation network or grain boundaries. Note that at high temperatures the static traps model for these impurities could become inaccurate, given the enhanced mobility of latter, however our whole model is designed to provide the qualitative analysis on the effect of impurities on loop diffusion. The comparable effect of C and O on loop diffusion and a much weaker effect of N atoms are expected to remain valid even if corrections for dynamic traps are necessary.

The available information of the concentration of H and He due to transmutation in plasma –facing components in DEMO after 5 years of operation^9^ gives us an additional possibility to estimate their effect on the loop diffusion (see data with square markers in Fig. 5). Here, one should also note, that unlike other impurities, for H and He the migration barrier is very low in the bulk W or vicinity of dislocation cores, being only of 0.06–0.09 eV^37,38^. Therefore, the application of static traps model is significantly limited in this case and offers a solely qualitative estimation, provided below, since both H and He are expected to be dragged by dislocation loops after attraction to them and even undergo the pipe diffusion along the line of dislocation loop^38^. In this case, assuming the limiting case of the absence of solute-loop drag effect, H does slow down the loop migration at the low temperature of 700 K and has no effect on it at the higher temperature of 1200 K. Meanwhile, He impurities in concentrations of 34 appm^9^ have potentially a tremendous effect on the loop mobility, exceeding by many orders of magnitude of the effects of the other interstitial impurities in the as-received concentration.

One should note that the irradiation-induced development of microstructure is assisted by a variety of many other processes which either stimulate the build-up of radiation defects (including not only dislocation loops but also voids, Re/Os precipitates^39^ etc.) or limit it. The latter includes processes such as the coalescence of vacancies into voids, SIAs and loops, absorption of loops into dislocation lines, segregation of substitutional solutes (Re/Os) to grain boundaries and into precipitates etc. In the limit of high irradiation neutron fluence, the diffusion of loops could be affected by additional factors^40–42^ such as: (1) interaction of loops with impurity-vacancy clusters^25,29,43,44^; (2) elastic interaction with other loops and grow-in dislocation network; (3) repulsive interaction with non-coherent precipitates, which basically “lock” 1D migrating loops preventing their long-range diffusion towards sinks; (4) thermal fluctuations of dislocations etc. The analysis presented in this work does not cover these physical phenomena.

## Conclusions

In conclusion, in this work we have performed ab initio simulations to calculate the interaction strength of the interstitial impurities, H, N, C, O and He, present in commercial W grades or created by neutron irradiation or injected from the plasma components or during ion irradiation, with self-interstitial and dislocation defects. Self-interstitial clusters of different size, starting from a single SIA and dislocation loops of three sizes (from 7 to 37 SIA, i.e. with a diameter 0.7–1.6 nm) up to a straight edge dislocation as a limiting case for an infinitely large dislocation loop were considered. Our analysis of the converged values of the impurity – loop interaction energy has shown that all the interstitial impurities have a strong affinity to the interstitial defects, with the interaction energy in the range from − 0.7 eV to − 2.2 eV. For the dislocation loops, the interaction energy ordering was the following: H < N < C < O < He. H is attracted significantly weaker to the loops compared to other impurities, while He exhibits the strongest attraction to the interstitial defects of any size.

In order to rationalize the interaction strength, i.e. different affinity of various impurities to the largest dislocation loop of 37 SIAs (1.7 nm in diameter), taken as an example, we have decomposed the interaction energy into elastic (due to minimization of lattice strain) and electronic (due to the decrease of impurity-neighbor W atoms interaction strength or, in other words, chemical bonding) contributions. For all the studied impurities, except for the N atom, the elastic contribution correlates with the magnitude of minimization of the impurity relaxation volume. The largest elastic contributions were identified for N, C and O atoms, with the highest value observed for the N atom, which was rationalized by a minimal increase of line length of dislocation loop upon insertion of this impurity as compared to other studied interstitial solutes. Naturally, the increase of the dislocation loop size (dislocation line length), observed for all studied impurities if allocated in the vicinity of the dislocation loop, implies an unfavorable energy cost to the system, reducing the elastic contribution to the interaction energy. The largest contribution from the minimization of the chemical bonding, analyzed by the evolution of net charge, bond order and sum of bond orders, was observed for H and He, which correlated with the change of a number of strong bonds and their weakening upon approaching the dislocation loop from the bulk by these impurities. No change in the type of chemical bond for any of the studied impurities with neighbor W atoms upon migration from the bulk to the vicinity of a large dislocation loop was observed, being of covalent character (with relatively large bond order and low net atomic charge) for all the cases.

Via a static trap model, we have also estimated the relative effect of the presence of various impurities on the mobility of isolated dislocation loops with 37 SIAs for different impurity concentrations and the two limiting temperatures of 700 and 1200 K relevant for DEMO operation. The modification of the loop migration process should have a significant effect on the evolution of the microstructure of the components, since any slowing down of the loop migration will mitigate their sinking at the dislocation network or grain boundaries. Thus, we derived that if C, N and O are dissolved in bulk W in their typical initial concentration, relevant for the beginning of the component operation, all these impurities will considerably affect the loop migration. It was noted that in this situation N atoms will affect the loop migration weaker than C and O impurities. The latter atoms will affect the loop mobility with a similar order of magnitude, accounting for their interaction energy with the loop (C is attracted weaker than O) and as-received concentration (more C than O atoms). The case of 5 years DEMO operation with H and He generation due to the nuclear transmutation reactions was also considered. It was shown that the transmuted H will affect the loop mobility only at the low temperature of 700 K, while He will have a tremendous effect at both 700 and 1200 K. However, given the low bulk or in-pipe dislocation core migration energy of H and He (< 0.1 eV), the static trap model is expected to break down in this case, and one can anticipate that H and He will diffuse with the loops as a single entity, thus limiting the trapping effect.

In conclusion, from the obtained results, C and O interstitial impurities are expected to have the largest impact on the loops’ diffusivities and hence have the largest impact on the evolution of the irradiation microstructure. Thus, our results suggest that the effects of both C and O should be accounted for coarse grain models like object kinetic Monte Carlo (OKMC) methods and rate theory models. The outcome of the nucleation of transmuted substitutional impurities including Re, Os, Ta etc. along with a full-scale OKMC simulation of the effect of complete relevant set of impurities on the mobility of dislocation loops at different doses for DEMO-relevant conditions will be in the focus of a follow-up work. Overall, the information on the atomic bonding and character of the interaction between different defects collected in this study has a strong potential for the transfer to upper scale models dealing with the assessment of the microstructural evolution under irradiation with such methods as atomistic or object kinetic Monte Carlo, dislocation dynamics and rate theory.

## Supplementary Information

Supplementary Information.
