# Supplementary material for: Impact of interstitial impurities on the trapping of dislocation loops in tungsten
Source: Sci Rep. 2021 Jun 10;11:12333. doi: 10.1038/s41598-021-91390-1 (PMC8192948; doi:10.1038/s41598-021-91390-1)
Supplement: Supplementary file 1 — Supplementary Information. [file 41598_2021_91390_MOESM1_ESM.docx]

Impact of interstitial impurities on the trapping of dislocation loops in tungsten

Alexander Bakaev, Giovanni Bonny, Nicolas Castin, Dmitry Terentyev, Viktor A. Bakaev

**Supplementary Material**

**Appendix A. Convergence study for the dislocation loop – impurity interaction**

For the reliable analysis of the interaction of interstitial impurities with dislocation loops a dedicated convergence study has been performed. It was important to follow it up given the large stress field introduced by the dislocation loops in the crystal and the unavoidable self-interaction through the periodic boundaries. Its effect was reduced by the application of the elastic correction (see Methodology section).

***Fig. A1.*** *Convergence study of the interaction of a carbon impurity with dislocation loops. ‘Gamma’ and ‘Monkh’ stand for gamma point and Monkhorst-pack k-point meshing, respectively.*

The convergence test was performed to compare two box sizes 8×8×8 and 9×9×9 and four different Bruilloin zone subdivisions (k-point meshes). The results have shown (see **Fig. A1**) that utilizing the converged k-point mesh (i.e., with the densest mesh of Monkhorst-pack 3×3×3) the difference in interaction energy carbon-dislocation loop between the box sizes 8×8×8 and 9×9×9 is less than 0.05 eV. For 9×9×9 box the accuracy of interaction energy values of 0.03 eV is already reached starting from k-point mesh of Monkhorst-pack 2×2×2. Thus, in order to obtain the accuracy of the results of dislocation-loop interaction of better than 0.05 eV complying with computational resources available we have chosen the parameterization of Monkhorst-pack 2×2×2 in a 9×9×9 simulation box.

**Appendix B. Stress tensor of pure W crystal for elastic correction of interaction energy and impurity relaxation volume calculations**

$\left( \begin{matrix} \begin{matrix} \begin{matrix} \sigma_{11} & \sigma_{12} \end{matrix} \\ \begin{matrix} \sigma_{21} & \sigma_{22} \end{matrix} \end{matrix} & \begin{matrix} \begin{matrix} \sigma_{13} & \sigma_{14} \end{matrix} \\ \begin{matrix} \sigma_{23} & \sigma_{24} \end{matrix} \end{matrix} & \begin{matrix} \begin{matrix} \sigma_{15} & \sigma_{16} \end{matrix} \\ \begin{matrix} \sigma_{25} & \sigma_{26} \end{matrix} \end{matrix} \\ \begin{matrix} \begin{matrix} \sigma_{31} & \sigma_{32} \end{matrix} \\ \begin{matrix} \sigma_{41} & \sigma_{42} \end{matrix} \end{matrix} & \begin{matrix} \begin{matrix} \sigma_{33} & \sigma_{34} \end{matrix} \\ \begin{matrix} \sigma_{43} & \sigma_{44} \end{matrix} \end{matrix} & \begin{matrix} \begin{matrix} \sigma_{35} & \sigma_{36} \end{matrix} \\ \begin{matrix} \sigma_{45} & \sigma_{46} \end{matrix} \end{matrix} \\ \begin{matrix} \begin{matrix} \sigma_{51} & \sigma_{52} \end{matrix} \\ \begin{matrix} \sigma_{61} & \sigma_{62} \end{matrix} \end{matrix} & \begin{matrix} \begin{matrix} \sigma_{53} & \sigma_{54} \end{matrix} \\ \begin{matrix} \sigma_{63} & \sigma_{64} \end{matrix} \end{matrix} & \begin{matrix} \begin{matrix} \sigma_{55} & \sigma_{56} \end{matrix} \\ \begin{matrix} \sigma_{65} & \sigma_{66} \end{matrix} \end{matrix} \end{matrix} \right)=\left( \begin{matrix} 532.28 & 203.86 & 203.86 & 0 & 0 & 0 \\ 203.86 & 532.28 & 203.86 & 0 & 0 & 0 \\ 203.86 & 203.86 & 532.28 & 0 & 0 & 0 \\ 0 & 0 & 0 & 138.29 & 0 & 0 \\ 0 & 0 & 0 & 0 & 138.29 & 0 \\ 0 & 0 & 0 & 0 & 0 & 138.29 \end{matrix} \right)$ MPa
